# Supplementary figures and images for: The Fiber Walk: A Model of Tip-Driven Growth with Lateral Expansion
Source: PLoS One. 2014 Jan 22;9(1):e85585. doi: 10.1371/journal.pone.0085585 (PMC3899046; doi:10.1371/journal.pone.0085585)

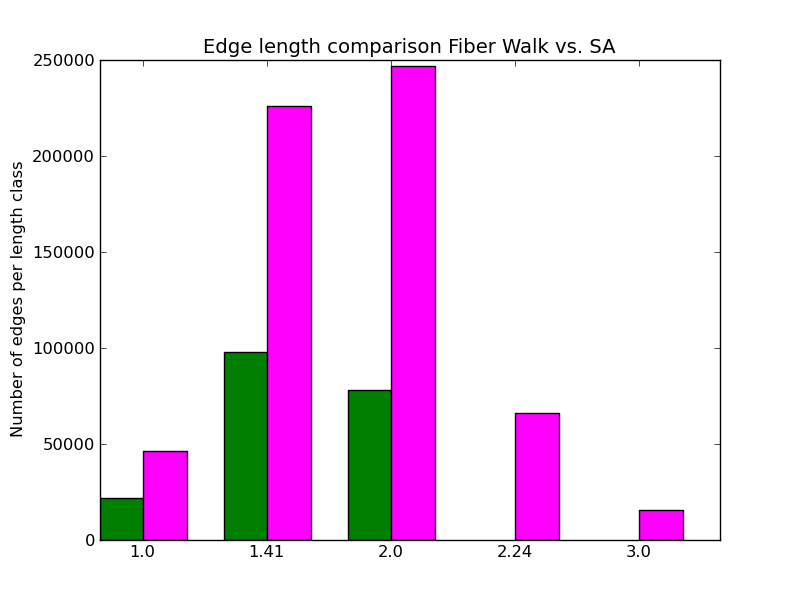

Supplement: Figure S1 — Comparison of edge length classes of the walk (green) and the self-avoiding edges on the lattice (purple) for the fiber walk in 2D. (TIFF) [file pone.0085585.s001.tiff]

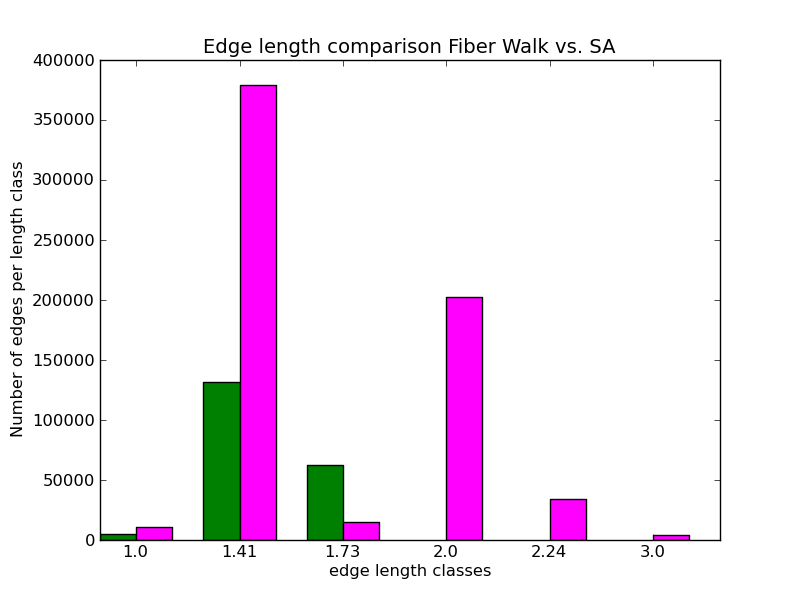

Supplement: Figure S2 — Comparison of edge length classes of the walk (green) and the self-avoiding edges on the lattice (purple) for the fiber walk in 3D. (TIFF) [file pone.0085585.s002.tiff]

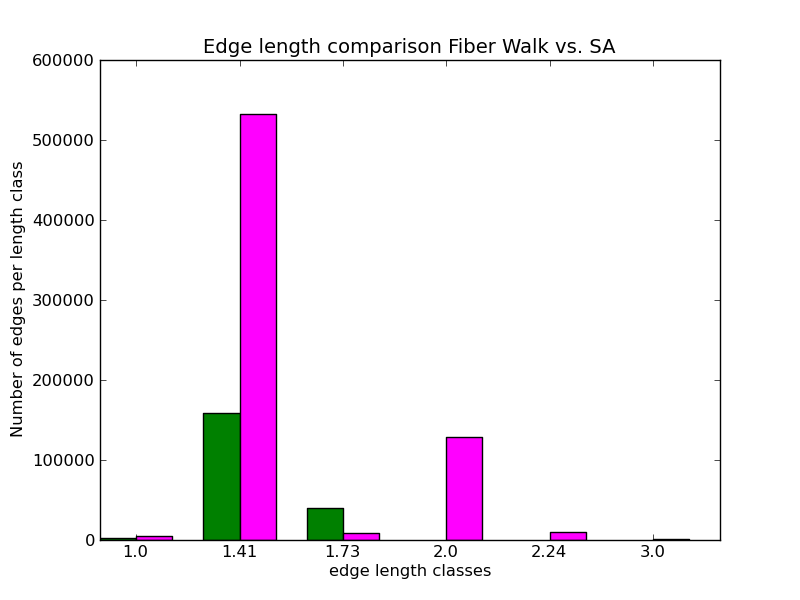

Supplement: Figure S3 — Comparison of edge length classes of the walk (green) and the self-avoiding edges on the lattice (purple) for the fiber walk in 4D. (TIFF) [file pone.0085585.s003.tiff]

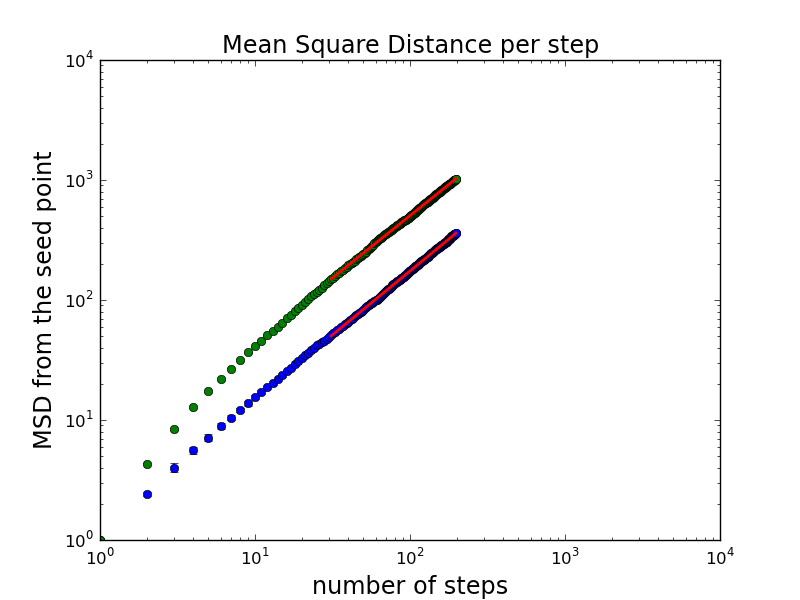

Supplement: Figure S4 — The average MSD scaling for the 3D growing SAW (blue) and the 3D fiber walk (green). The scaling exponent for the growing SAW is and for the fiber walk based on 1000 walks of length 200. (TIFF) [file pone.0085585.s004.tiff]

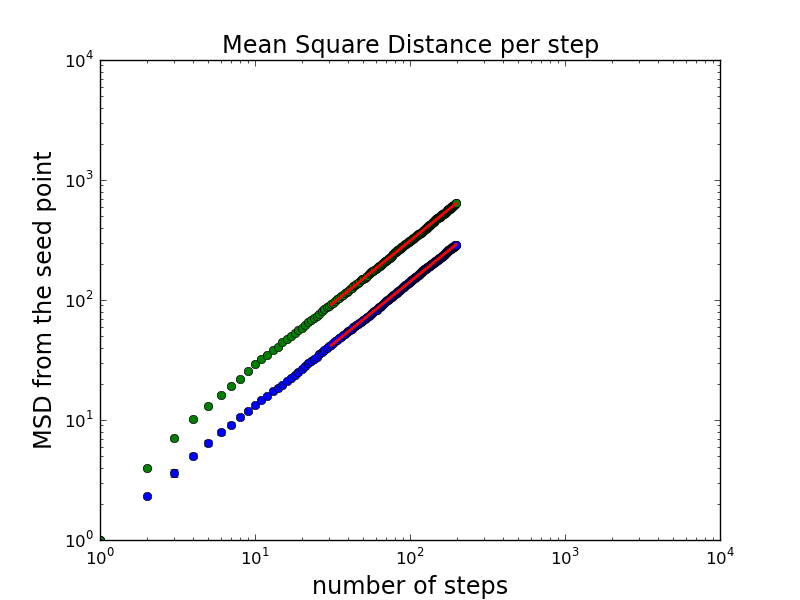

Supplement: Figure S5 — The average MSD scaling for the 4D growing SAW (blue) and the 3D fiber walk (green). The scaling exponent for the growing SAW is and for the fiber walk based on 1000 walks of length 200. (TIFF) [file pone.0085585.s005.tiff]

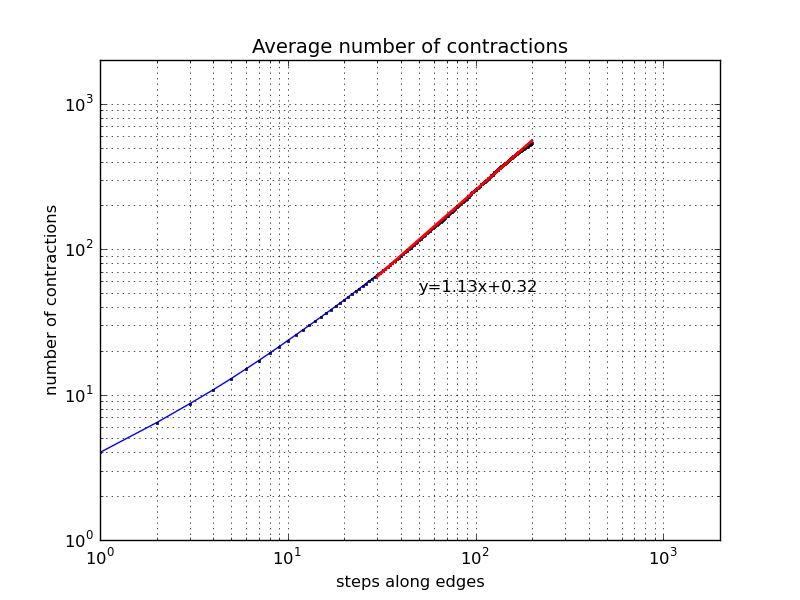

Supplement: Figure S6 — The number of contractions indicating the size of the area enclosed by the region for the 2D fiber walk resulting in the scaling exponent of 1.13 for 1000 walks of length 200. (TIFF) [file pone.0085585.s006.tiff]

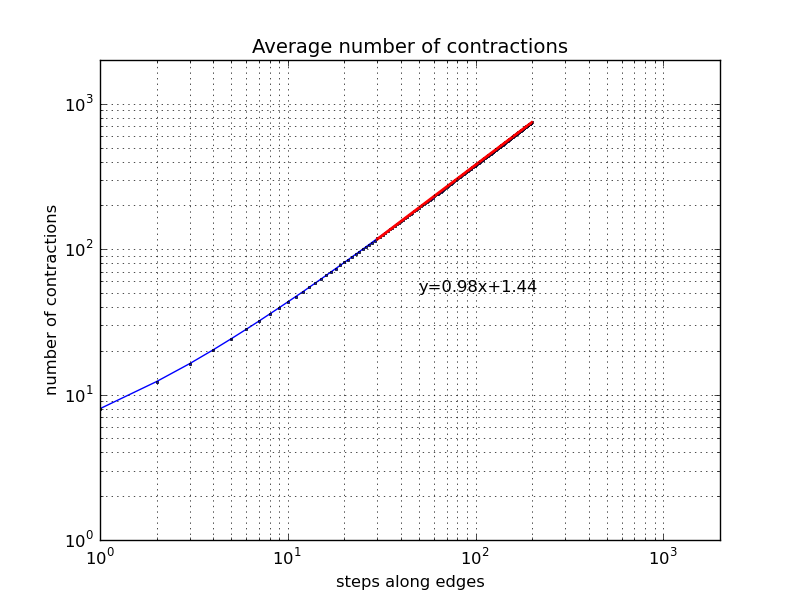

Supplement: Figure S7 — The number of contractions indicating the size of the area enclosed by the region for 3D fiber walk resulting in the scaling exponent of 0.98 for 1000 walks of length 200. (TIFF) [file pone.0085585.s007.tiff]

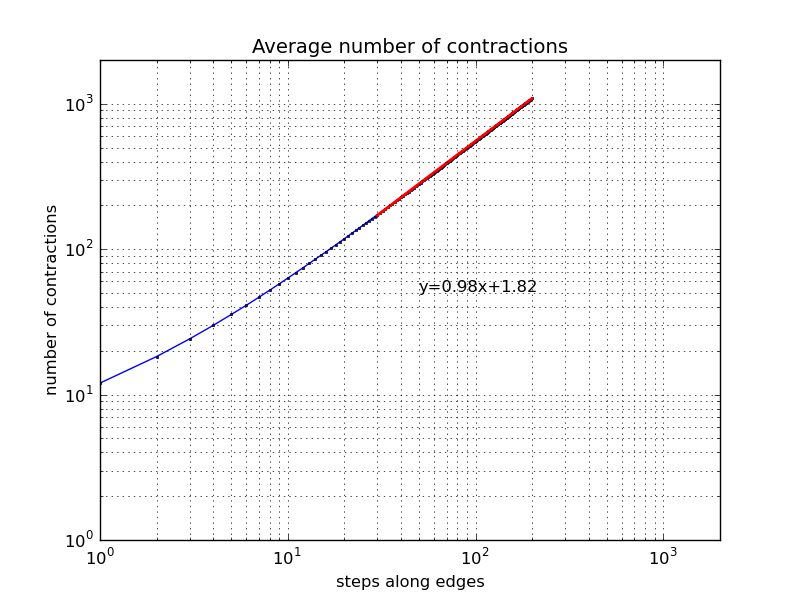

Supplement: Figure S8 — The number of contractions indicating the size of the area enclosed by the region for the 4D fiber walk resulting in the scaling exponent of 0.98 for 1000 walks of length 200. (TIFF) [file pone.0085585.s008.tiff]
